# Atractylodis Rhizoma Alba Attenuates Neuroinflammation in BV2 Microglia upon LPS Stimulation by Inducing HO-1 Activity and Inhibiting NF-κB and MAPK

**DOI:** 10.3390/ijms20164015

**Published:** 2019-08-17

**Authors:** Yun Hee Jeong, Wei Li, Younghoon Go, You-Chang Oh

**Affiliations:** Korean Medicine (KM)-Application Center, Korea Institute of Oriental Medicine, Daegu 41062, Korea

**Keywords:** Atractylodis Rhizoma Alba (ARA), anti-neuroinflammation, heme oxygenase (HO)-1, nuclear factor (NF)-κB, mitogen-activated protein kinases (MAPK)

## Abstract

Microglial activation and the resulting neuroinflammation are associated with a variety of brain diseases, such as Alzheimer’s disease and Parkinson’s disease. Thus, the control of microglial activation is an important factor in the development of drugs that can treat or prevent inflammation-related neurodegenerative disorders. Atractylodis Rhizoma Alba (ARA) has been reported to exhibit antioxidant, gastroprotective, and anti-inflammatory effects. However, the effects of ARA ethanolic extract (ARAE) on microglia-mediated neuroinflammation have not been fully elucidated. In this work, we explored the anti-neuroinflammatory properties and underlying molecular mechanisms of ARAE in lipopolysaccharide (LPS)-stimulated microglial BV2 cells. Our results showed that ARAE significantly attenuates the production of nitric oxide (NO) and inflammatory cytokines induced by LPS. ARAE treatment also inhibited the expression of inducible nitric oxide synthase (iNOS) and cyclooxygenase (COX)-2 without causing cytotoxicity. ARAE markedly attenuated the transcriptional activities of nuclear factor (NF)-κB and mitogen-activated protein kinases (MAPK) phosphorylation, and induced heme oxygenase (HO)-1 expression. High-performance liquid chromatography (HPLC) analysis showed that ARAE contains three main components—atractylenolide I, atractylenolide III, and atractylodin—all compounds that significantly inhibit the production of inflammatory factors. These findings indicate that ARAE may be a potential therapeutic agent for the treatment of inflammation-related neurodegenerative diseases.

## 1. Introduction

Microglia are major resident immune cells and play an important role in the host defense recovery of the central nervous system (CNS) as macrophages in the brain. In their unstimulated state, microglia are constantly scavenging the CNS for damaged neurons and pathogens, and also act to maintain synaptic homeostasis [1]. However, sustained excessive activation of microglia leads to neuronal damage and the release of neurotoxic substances, including nitric oxide (NO), tumor necrosis factor (TNF)-α, interleukin (IL)-6, IL-1β, inducible nitric oxide synthase (iNOS), and cyclooxygenase (COX)-2, all of which are implicated in neurodegenerative disorders including Alzheimer’s disease, Parkinson’s disease, and stroke [2,3]. Many studies are therefore underway to develop novel agents which can treat brain diseases by attenuating the secretion of pro-inflammatory mediators through the regulation of over-activated microglia.

Lipopolysaccharides (LPS) act as prototypical endotoxins, inducing inflammation, septic shock, and death. They are therefore commonly used as an in vitro model of inflammation [4,5]. During the progression of inflammatory processes activated by LPS, nuclear factor (NF)-κB and mitogen-activated protein kinases (MAPK) have been regarded as typical inflammatory signaling pathways. The members of the MAPK family, including extracellular signal-regulated kinase (ERK), c-Jun NH_2_-terminal kinase (JNK) and p38, are related to the regulation of the secretion of inflammatory factors in stimulated microglia [6]. This signaling pathway in turn mediates transcription factors such as NF-κB. NF-κB consists of p65/p50 subunits in mammalian cells and is a pivotal transcription factor. When activated, NF-κB is translocated to the nucleus and promotes the expression of several inflammatory mediators. These molecules include iNOS and COX-2, and pro-inflammatory cytokines in microglia [7,8]. Thus, the MAPK and NF-κB pathways are considered to be important potential targets for the treatment of neuroinflammatory disease. Heme oxygenase (HO)-1 is a rate-limiting enzyme that catalyzes the degradation of the heme group to carbon monoxide (CM), biliverdin, and free iron. HO-1 and its by-product CM decreases inflammatory cytokines, iNOS, and COX-2 production, thereby reducing the level of iNOS-derived NO and COX-2-derived prostaglandin (PG) E_2_ [9,10,11]. Therefore, many therapeutic agents present their anti-neuroinflammatory effects through enhancement of HO-1 expression [12,13].

Atractylodis Rhizoma Alba (ARA) is the dried root stock of *Atractylodis japonica* Koidzumi or *Atractylodes macrocephala* Koidzumi, known as “Sabju” or “Baekchul” in Korea. ARA has had a long history of use as a medicinal herb to treat loss of appetite and abdominal distention in East Asia. In addition, ARA has been used to treat systemic edema caused by water excretion disorders, diarrhea, coughing, cold sweats, and morning sickness. In traditional oriental medicine, ARA is known for its sweet and bitter taste and warm nature, and the shape of the herb is in the form of uneven lumps or an uneven curved circumference and is yellow and white in color. Previous studies have reported that ARA has a range of pharmacological functions, including antioxidant, gastroprotective, and anti-inflammatory effects [14,15]. In a past study, n-hexane extracts from ARA and its constituents have been reported to be effective in suppressing the inflammatory response of RAW 264.7 macrophages [14], however, the activity and molecular mechanisms of microglia-mediated anti-neuroinflammatory effects generated by ARA ethanolic extract (ARAE) still remain unknown. Several previous studies using ARA also examined the regulatory effects for in vitro inflammatory reactions, but none using neuroinflammatory models associated with neurodegenerative diseases. Therefore, we used a brain-specific macrophage BV2 cell line to induce neuroinflammatory reactions, and by investigating the effects of ARAE on it, we studied whether ARAE has the potential control ability for neurodegenerative diseases. The purpose of this study was to investigate the anti-neuroinflammatory effects of ARAE and to clarify how ARAE changes molecular mechanisms such as NF-κB, MAPK, and HO-1 to indicate such efficacy. We also investigated the constituents of ARAE using HPLC analysis.

## 2. Results

### 2.1. Effects of ARAE on the Viability of BV2 Microglia

**Figure 1:**
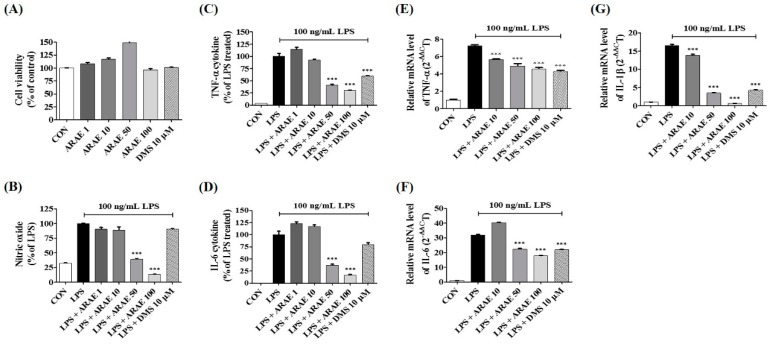
Effects of ARAE on (**A**) cell viability, (**B**) nitric oxide (NO) secretion, (**C**,**D**) inflammatory cytokines production, and (**E**–**G**) mRNA expression in BV2 microglia. Cells were seeded on a culture plate and pre-incubated for 18 h. Control cells were incubated with vehicle alone. Data represent the mean ± standard error of the mean (SEM) of duplicate determinations from three independent experiments. CON: control; LPS: lipopolysaccharide; ARAE: Atractylodis Rhizoma Alba ethanolic extract; DMS: dexamethasone. *** *p* < 0.0001.

To examine the cytotoxic effects of ARAE on BV2 microglial cells, cell-counting kit (CCK) assays were performed. As shown in Figure 1A, treatment with ARAE alone for 24 h at no more than 100 μg/mL produced no significant changes in cell viability compared to a non-treated control group. Therefore, we used concentrations at 100 μg/mL or below for all subsequent experiments.

### 2.2. Effects of ARAE on Secretion of NO and Production of Inflammatory Cytokines

To measure the anti-neuroinflammatory activity of ARAE, we evaluated the production of neuroinflammatory factors. We first assessed the inhibitory effect of ARAE on the level of NO in LPS-stimulated BV2 cells. The level of NO released into the culture medium was analyzed using Griess reagent. As shown in Figure 1B, LPS stimulation dramatically enhanced the levels of NO compared to non-treated controls. However, pretreatment of the cells with ARAE strongly diminished NO production in a concentration-dependent manner. Cytokines are known to play a critical role in inflammatory effects on LPS-stimulated neuroinflammation and neuronal function [16,17]. We examined the effects of ARAE on LPS-stimulated inflammatory cytokine production and their mRNAs in BV2 microglial cells, using enzyme-linked immunosorbent assay (ELISA) and real-time reverse transcription-polymerase chain reaction (real-time RT-PCR), respectively. As shown in Figure 1C,D, LPS alone significantly increased the expression of TNF-α and IL-6 cytokines, while ARAE treatment markedly suppressed cytokine production in a concentration-dependent manner. Pretreatment with ARAE resulted in significant concentration-dependent reductions in TNF-α, IL-6, and IL-1β mRNA levels, consistent with previous results, as shown in Figure 1E–G.

### 2.3. Effects of ARAE on the Expression of iNOS and COX-2 and Induction of HO-1

**Figure 2:**
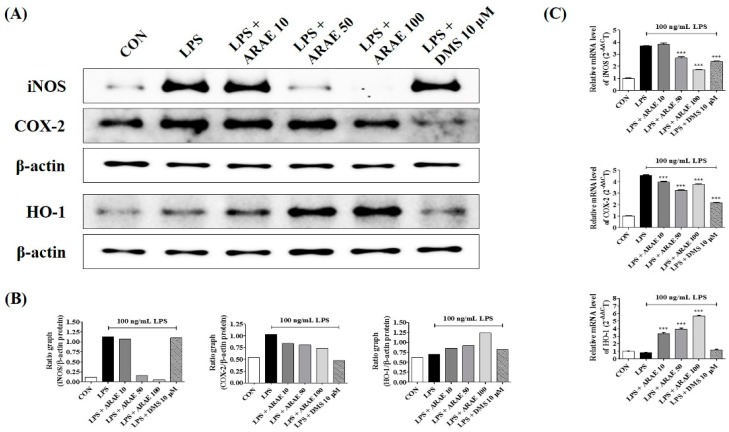
Effects of ARAE on (**A**,**B**) protein and (**C**) mRNA expression of inducible nitric oxide synthase (iNOS), cyclooxygenase (COX)-2, and heme oxygenase (HO)-1. Cells were seeded on a culture plate and pre-incubated for 18 h. Control cells were incubated with vehicle alone. Microglia were stimulated with LPS for 12 h (iNOS and COX-2), 6 h (HO-1 protein), or 3 h (HO-1 mRNA). (**B**,**C**) The histograms show protein and mRNA expression levels relative to those of the internal control. (**C**) Data represent the mean ± SEM of three independent experiments. CON: control; LPS: lipopolysaccharide; ARAE: Atractylodis Rhizoma Alba ethanolic extract; DMS: dexamethasone. *** *p* < 0.0001.

NO and PGE_2_ are synthesized by iNOS and COX-2, respectively. We used western blotting and real-time RT-PCR analysis to detect the expression levels of iNOS and COX-2 in BV2 microglia upon LPS stimulation. The data indicated that pretreatment with ARAE greatly suppressed the production of iNOS and COX-2 stimulated by LPS at both protein and mRNA levels in a concentration-dependent manner, as shown in Figure 2. We evaluated the efficacy of ARAE for HO-1 induction in BV2 cells, at both protein and mRNA levels. As shown in Figure 2, ARAE treatment significantly increased the expression of HO-1 protein in a concentration-dependent manner. Also, according to real-time RT-PCR analysis, ARAE treatment induced the expression of HO-1 mRNA at a statistically significant level.

### 2.4. Effects of ARAE on Transcriptional Activity of NF-κB in LPS-Stimulated BV2 Microglia

**Figure 3:**
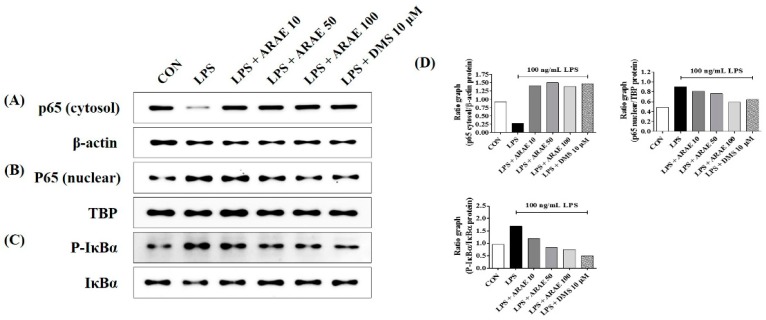
Effects of ARAE on nuclear factor (NF)-κB p65 (**A**) cytosol, (**B**) nuclear protein expression, and (**C**) phosphorylation and degradation of inhibitor of NF-κB alpha (IκBα). Cells were seeded on a culture plate and pre-incubated for 18 h. Control cells were incubated with vehicle alone. Cells were stimulated with LPS for (**A**,**B**) 1 h or (**C**) 30 min. (**D**) The histograms show protein expression levels relative to those of the internal control. CON: control; LPS: lipopolysaccharide; ARAE: Atractylodis Rhizoma Alba ethanolic extract; DMS: dexamethasone.

The NF-κB signaling pathway is well known to play an important role in modulating inflammatory responses. We investigated the inhibitory effects of ARAE on inhibitor of NF-κB alpha (IκBα) phosphorylation and transactivation of NF-κB in LPS-stimulated BV2 cells. Western blotting showed that pretreatment with ARAE strongly inhibited translocation of the p65 subunit of NF-κB from the cytoplasm into the nucleus in a concentration-dependent manner, as shown in Figure 3A,B,D. Additionally, as shown in Figure 3C,D, ARAE significantly decreased the LPS-induced degradation and phosphorylation of IκBα in a concentration-dependent manner. These findings suggest that ARAE prevents neuroinflammation through the inhibition of NF-κB signaling activation.

### 2.5. Effects of ARAE on the Activation of MAPK Induced by LPS in BV2 Microglia

**Figure 4:**
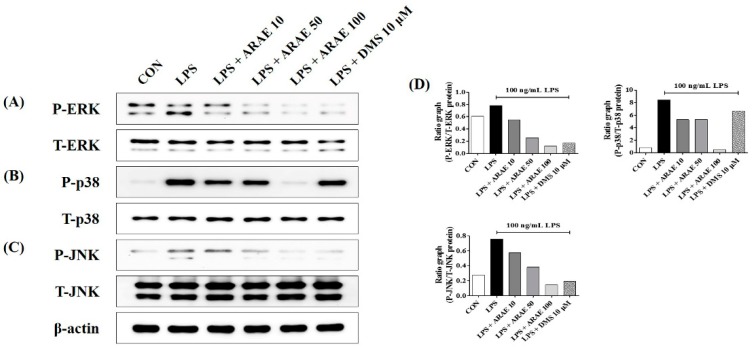
Effects of ARAE on phosphorylation of (**A**) extracellular signal-regulated kinase (ERK), (**B**) p38, and (**C**) c-Jun NH_2_-terminal kinase (JNK) mitogen-activated protein kinases (MAPK). Cells were seeded on a culture plate and pre-incubated for 18 h. Control cells were incubated with vehicle alone. Cells were stimulated with LPS for 30 min. (**D**) The histograms show protein expression levels relative to those of total-type protein. CON: control; LPS: lipopolysaccharide; ARAE: Atractylodis Rhizoma Alba ethanolic extract; DMS: dexamethasone.

The phosphorylation of three MAPK family proteins—ERK, p38, and JNK—is known to be involved in the expression of various pro-inflammatory mediators via the activation of transcription factor NF-κB [18,19]. We measured the repression of the activation of ERK, p38, and JNK in LPS-stimulated BV2 microglia. Our results indicated that ARAE treatment inhibited phosphorylation of all members of the MAPK family, including ERK, p38, and JNK, as shown in Figure 4.

### 2.6. Identification of the Components of ARAE Using HPLC-Diode Array UV/VIS Detector (DAD) Analysis

**Figure 5:**
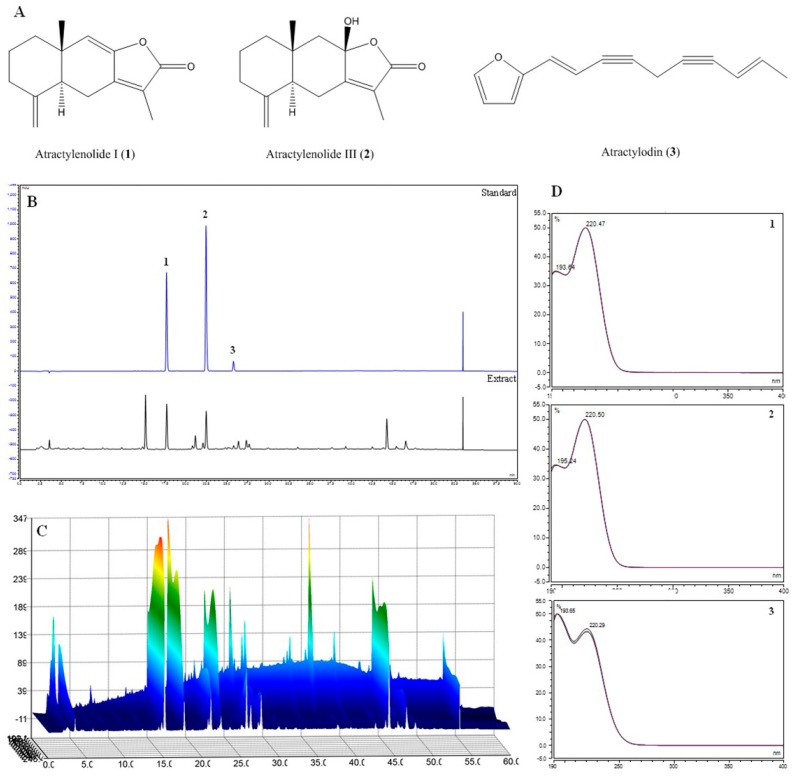
Structure of three major compounds (**A**), high-performance liquid chromatography (HPLC) chromatograms (**B**: 2D; **C**: 3D) of three standard compounds from the ARAE at UV wavelengths of 220 nm (**D**). Atractylenolide I (**1**), atractylenolide III (**2**), and atractylodin (**3**) were identified. ARAE: Atractylodis Rhizoma Alba ethanolic extract.

We used HPLC-DAD analysis, with the mobile phase consisting of acetonitrile (ACN) and water, and a C18 column as the stationary phase. Analytes were detected at wavelengths of 200–400 nm using a photodiode array (PDA) detector to examine multiple classes of compounds. A wavelength of 220 nm was selected for detection. As shown in Figure 5, based on comparison of chromatograms with each standard, retention times (tR), and UV spectra, the standards had retention times which included atractylenolide I (1; 17.66 min), atractylenolide III (2; 22.42 min), and atractylodin (3; 25.73 min). All standards showed good selectivity without interference within 40 min.

### 2.7. Validation of the Analytical HPLC Method

The calibration curves for the three major compounds were obtained by plotting the peak areas against the concentration using least-squares regression analysis. Each calibration equation was acquired from five concentrations ranging from 12.5 to 200 μg/mL. The linear correlation coefficient (*R*^2^) for the calibration curves was greater than 0.99, indicating a strong linear relationship, as shown in Table 1. From the calibration curves, the amounts of compounds 1–3 were found to be 16.04287 ± 0.0175, 10.39714 ± 0.0712, and 17.06388 ± 0.0702 mg/g, respectively.

### 2.8. Verification of Neuroinflammatory Inhibitory Efficacy of Atractylenolide I, Atractylenolide III, and Atractylodin in LPS-Stimulated BV2 Microglia

**Figure 6:**
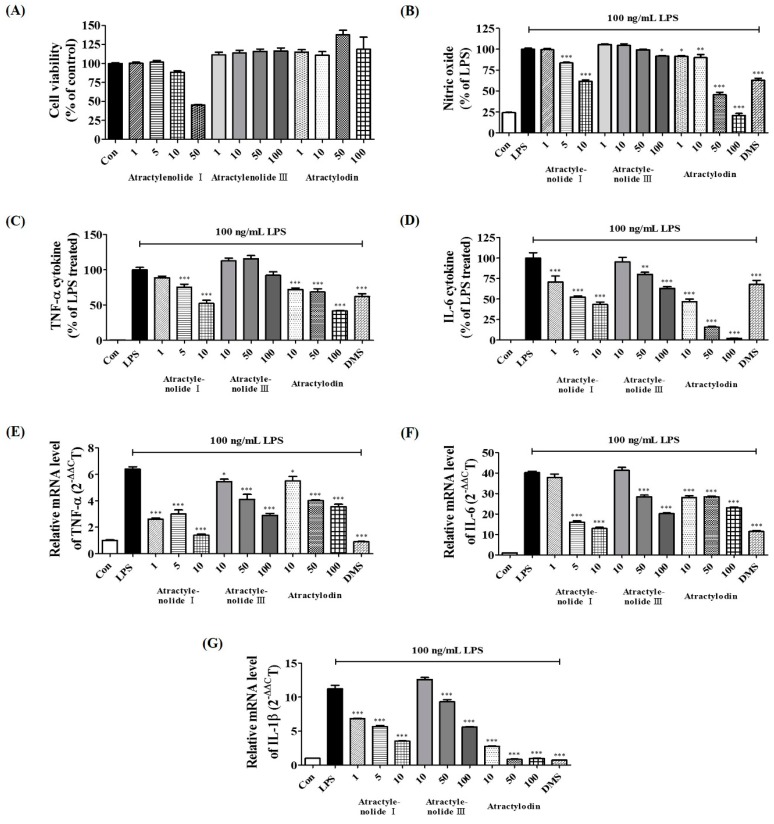
Effects of three compounds—atractylenolide I, atractylenolide III, and atractylodin—on (**A**) cell viability and the production of (**B**) NO, (**C**) tumor necrosis factor (TNF)-α, (**D**) interleukin (IL)-6 cytokines, and (**E**–**G**) cytokine mRNAs. Cells were seeded on a culture plate and pre-incubated for 18 h. Control cells were incubated with vehicle alone. Data represent the mean ± SEM of duplicate determinations from three independent experiments. Con: control; LPS: lipopolysaccharide; DMS: 10 μM dexamethasone. * *p* < 0.05; ** *p* < 0.001; *** *p* < 0.0001.

To confirm the cytotoxic effect of the main constituents of ARAE, we performed CCK assays on BV2 microglia cells. Results showed that treatment with atractylenolide I at no more than 10 μM, and atractylenolide III, and atractylodin at no more than 100 μM had no influence on cell viability, as shown in Figure 6A. Therefore, the subsequent experiments were carried out at concentrations within the non-cytotoxic range for each drug. We first investigated the effects of three compounds of ARAE on the production of inflammatory mediators. As shown in Figure 6B,C, all compounds, except for atractylenolide III, showed significant inhibition of the production of NO and TNF-α in a concentration-dependent manner. In addition, all compounds showed marked concentration-dependent diminution of the production of cytokine IL-6, as shown in Figure 6D. Next, we analyzed the influence of three compounds on the expression of each cytokine mRNA. TNF-α mRNA was inhibited in a pattern similar to cytokine secretion by three compounds, and atractylenolide III also showed a concentration-dependent inhibitory effect, as shown in Figure 6E. Expression of IL-6 mRNA, like cytokine level, was significantly inhibited by all compounds, as shown in Figure 6F. The three components of ARAE showed the best inhibitory activity for IL-1β mRNA expression, especially atractylodin which showed very strong inhibitory effect at all concentrations, as shown in Figure 6G.

## 3. Discussion

Microglia-mediated uncontrolled neuroinflammation contributes to the progress of neurodegenerative diseases such as Alzheimer’s disease, Parkinson’s disease, and stroke. Excessive activation of microglia leads to neuronal death, brain injury, and the release of a variety of neuroinflammation mediators such as NO, iNOS, COX-2, TNF-α, IL-6, and IL-1β [20].

The reduction of these endogenous inflammatory factors and toxic cytokines via modulation of the activation of microglia is important in the prevention and treatment of neuroinflammation [21]. ARA is an important traditional medicine that is commonly used in treatment of loss of appetite and abdominal distention in Korea. Previous studies have reported several pharmacological functions of ARA, such as preventing obesity and glucose intolerance [22] and managing type 2 diabetes via promoting the differentiation of adipocytes [23]. However, the effects of ARAE on microglia-mediated neuroinflammatory and inhibitory mechanisms have not been explored. In this study, we identified anti-neuroinflammatory effects and molecular mechanisms of ARAE in LPS-stimulated BV2 cells. NO is a free radical that is known to be implicated in the process of microglia-mediated inflammation in the CNS [24]. NO is synthesized from iNOS, and overproduction of NO is correlated with the presence of several inflammatory and autoimmune diseases [25,26]. COX-2 is related to activating macrophages or microglia by inflammatory stimuli such as LPS and is a synthetic enzyme of PGE_2_ and plays an important role in maintaining and transmitting inflammatory reactions [7]. We examined whether ARAE pretreatment inhibited the secretion of NO during LPS-stimulated neuroinflammation in BV2 microglia. Our results showed that ARAE dramatically reduced NO expression and strongly reduced both the protein and mRNA levels of iNOS and COX-2 in a concentration-dependent manner. Because the NO and iNOS levels were closely associated with the induction of HO-1 [27], we further determined the influence of ARAE treatment on HO-1 levels in LPS-stimulated microglial BV2 cells. We found that ARAE markedly induced HO-1 protein and mRNA expression in a concentration-dependent manner. Moreover, ARAE significantly decreased the secretion of pro-inflammatory cytokines and the expression of their mRNA genes, including TNF-α, IL-6, and IL-1β, in a dose-dependent manner.

The activation of the NF-κB and MAPK signaling pathways has been associated with the pathogenesis of various diseases of the CNS. NF-κB transcription factors are key regulators of a variety of genes involved in immune and inflammatory responses [28]. In unstimulated cells, p65 of NF-κB functions as part of a complex with its suppressor protein IκBα in the cytoplasm. Upon stimulation of microglial cells by inflammatory stimulants such as LPS, IκBα is phosphorylated and degraded, which then frees NF-κB to translocate into the nucleus to promote pro-inflammatory factors [29]. We investigated whether the inhibitory effects of ARAE on pro-inflammatory factors are present throughout the NF-κB pathway. Our results indicated that ARAE treatment significantly inhibited nuclear migration of NF-κB p65 in LPS-stimulated microglia BV2 cells via suppression of IκBα degradation and phosphorylation in a concentration-dependent manner. The MAPK pathway plays an important role in the LPS-induced expression of several endogenous inflammatory mediators, as well as in the activation of NF-κB [30,31]. We assessed the efficacy of ARAE on the phosphorylation of the MAPK family proteins, ERK, p38, and JNK by LPS stimulation, which indicated that the activation of three MAPK proteins was effectively attenuated by pretreatment with ARAE. These results suggested that the anti-neuroinflammatory effects of ARAE might be regulated through the inhibition of NF-κB and the suppression of MAPK.

We also identified the main components (atractylenolide I, atractylenolide III, and atractylodin) of the ARAE, using HPLC analysis, as shown in Figure 5. Previous studies of these components have shown that atractylenolide I appears to have a neuroprotective role in a Parkinson’s disease model [32]. There have also been reports that atractylenolide III inhibits the activation of NF-κB and MAPK in LPS-stimulated macrophages [33]. Atractylodin has also been reported to be effective in inhibiting LPS-induced activation of the NLRP3 inflammasome and TLR4 pathways [34]. These studies indicated that the anti-neuroinflammatory efficacy of ARAE is closely related to the activity of its constituents, atractylenolide I, atractylenolide III, and atractylodin. In order to confirm the anti-neuroinflammatory activity of these components, we evaluated the effect of atractylenolide I, atractylenolide III, and atractylodin on the production of NO, inflammatory cytokines, and cytokine mRNAs on the microglia, and confirmed that the compounds showed anti-neuroinflammatory activity. Our study indicated that all of these compounds, except for atractylenolide III, inhibited the production of NO and TNF-α cytokine, as shown in Figure 6B,C. Also, all compounds suppressed IL-6 cytokine production, as shown in Figure 6D. In addition, the three compounds also showed excellent inhibitory effect for expression of each cytokine mRNA, and strongly inhibited the expression of IL-1β mRNA, as shown in Figure 6E–G.

## 4. Materials and Methods

### 4.1. Materials and Reagents

Dulbecco’s modified Eagle’s medium (DMEM), fetal bovine serum, and antibiotics were obtained from Hyclone (Logan, UT, USA). LPS, dexamethasone (DMS), bovine serum albumin (BSA), and dimethyl sulfoxide (DMSO) were purchased from Sigma–Aldrich (St. Louis, MO, USA). ELISA antibody sets were obtained from eBioscience (San Diego, CA, USA). Cell culture dishes and plates were purchased from Sarstedt (Nümbrecht, Germany). A CCK was obtained from Dojindo Molecular Technologies, Inc. (Kumamoto, Japan). Primary antibodies and horseradish peroxidase (HRP)-conjugated secondary antibodies were purchased from Cell Signaling Technology, Inc. (Boston, MA, USA). An RNA extraction kit was obtained from iNtRON Biotech (Daejeon, Korea). DNA synthesizing kits and an AccuPower^®^ 2× Greenstar qPCR Master Mix were obtained from Bioneer (Daejeon, Korea). Oligonucleotide primers for real-time RT-PCR were synthesized by Bioneer. ARAE was obtained as a freeze-dried powder from the Korea Plant Extract Bank (Ochang, Korea), dissolved in dimethyl sulfoxide (DMSO) and stored at −20 °C before use. Chemical standards for ARAE (atractylenolide I, atractylenolide III, and atractylodin) were purchased from ChemFaces (Wuhan, China).

### 4.2. Cell Culture and Drug Treatment

BV2 cells were obtained from Professor Kyoungho Suk at Kyungpook National University (Daegu, Korea) and grown in complete DMEM. The cells were then incubated with humidified 5% CO_2_ at 37 °C. To stimulate the cells, 100 ng/mL LPS was added at the indicated periods in the presence or absence of ARAE (10, 50, or 100 μg/mL) or DMS (10 μM).

### 4.3. Cell Viability Test

Cytotoxicity induced by ARAE was analyzed using a CCK. BV2 cells were seeded into 96-well plates at a density of 5 × 10^4^ cells/well. After 18 h of incubation, ARAE was added to the cells, which were incubated for 48 h at 37 °C with 5% CO_2_. Treatments of CCK solution, incubation time, and analysis methods were performed according to a previous study, with some modifications [35].

### 4.4. Determination of NO Production

NO production was analyzed by measuring the nitrite levels in the supernatants of cultured macrophages. BV2 cells (5 × 10^4^ cells/well) were plated, incubated with ARAE, and stimulated with LPS for 24 h. Griess reagent treatment and analysis were performed as previously reported [35]. The concentration of nitrite was calculated using sodium nitrite as the standard.

### 4.5. Cytokine Determination

To determine the effects of ARAE on the production of pro-inflammatory cytokines, cytokine production was measured using ELISA. For ELISA, 2.5 × 10^5^ BV2 cells/well were seeded into 24-well plates and incubated overnight. The cells were pretreated with various concentrations of ARAE for 1 h and further challenged with LPS for an additional 24 h at 37 °C under 5% CO_2_ [35]. The levels of cytokines in the supernatants were measured using ELISA antibody sets, according to the manufacturer’s protocol.

### 4.6. Preparation of Whole Cell, Cytosolic, and Nuclear Extracts

To obtain whole cell lysates, pellets were resuspended in radioimmunoprecipitation assay lysis buffer (Millipore, Bedford, MA, USA) containing protease and phosphatase inhibitors. Cytosolic and nuclear fractions were isolated using NE-PER^TM^ nuclear and cytoplasmic extraction reagents (Thermo Scientific, Rockford, IL, USA) as described by the manufacturer. The fractions were stored at −80 °C before use.

### 4.7. Western Blot Analyses

Total proteins of whole cell lysates or cytoplasmic and nuclear extracts were assayed using Bradford’s reagent (Bio-Rad, Hercules, CA, USA). The proteins were divided using sodium dodecyl sulfate-polyacrylamide gel electrophoresis (SDS-PAGE) and transferred to a nitrocellulose (NC) membrane (Millipore, Bedford, MA, USA). After blocking of nonspecific sites with 3% BSA, membranes were incubated with each primary antibody at room temperature (RT) for 2 h or at 4 °C overnight. The membranes were subsequently incubated with HRP-conjugated anti-mouse or anti-rabbit secondary antibodies. Signals were detected using Super Signal West Femto chemiluminescent substrate (Thermo Scientific, Rockford, IL, USA). Protein levels were quantified using a ChemiDoc^TM^ Touch Imaging System (Bio-Rad, Hercules, CA, USA).

### 4.8. Total RNA Extraction and RT-PCR

Total cellular RNA was extracted using easy-BLUE^™^ RNA extraction kits (iNtRON Biotech, Daejeon, Korea) in accordance with the manufacturer’s protocol. cDNA was synthesized from 1 μg of total RNA using AccuPower^®^ CycleScript RT PreMix (Bioneer, Daejeon, Korea). The oligonucleotide primers for real-time RT-PCR are indicated in Table 2 [36]. The samples were setup in triplicate in a 20 μL total volume: 0.3 μM final concentrations of each primer (1 μL of forward and reverse primer), 10 μL of AccuPower^®^ 2× Greenstar qPCR Master Mix (Bioneer, Daejeon, Korea), 3 μL of 0.1% DEPC-treated water, and 5 μL of template DNA. The following PCR conditions were applied: TNF-α, IL-6, IL-1β, iNOS, COX-2, HO-1, and β-actin, with 40 cycles of 94 °C for 15 s and 60 °C for 1 min [36]. The amplification and analysis were performed using a QuantStudio 6 Flex Real-time PCR System (Thermo Scientific, Rockford, IL, USA). Samples were compared using the relative *C*t method. The results of real-time RT-PCR were presented as inflammatory mediator gene induction fold, which were calculated using β-actin as the internal control.

### 4.9. Sample Preparation for HPLC Analysis

All sample solutions were dissolved in methanol. Standard stock solutions of compounds were prepared at a concentration of 1 mg/mL and were then serially diluted with methanol to obtain a calibration curve of standard solutions at various concentration levels ranging from 12.5–200 μg/mL for all three compounds. ARAE extract was prepared at a concentration of 5 mg/mL. All solutions for analysis were filtered through 0.45 μm regenerated cellulose (RC) membrane syringe filters (Sartorius, Germany).

### 4.10. Optimization of Chromatographic Conditions

HPLC analysis was performed using a Dionex UltiMate 3000 system (Dionex Corp., Sunnyvale, CA, USA) equipped with a binary pump, auto-sampler, column oven, and DAD. System control and data analysis were carried out using Dionex Chromelon software. Separation was carried out on a Luna C18 column (250 × 4.6 mm, 5 μm, Phenomenex, Torrance, CA, USA), with the column oven temperature kept at 30 °C, at a UV wavelength of 220 nm. The mobile phase consisted of water (solvent A) and acetonitrile (solvent B) with gradient elution of 0–40 min, 40%–100% B at a flow rate of 1.0 mL/min. Before injection of the next sample, the column was re-equilibrated with the initial gradient of solvents for 10 min.

### 4.11. Validation of the Method

Specificity was assessed by comparing the chromatographic profile of the standard with that of the sample extract. Linearity was assessed by computing the correlation coefficient (*R*^2^) of the calibration curve for each compound at concentrations ranging from 12.5 to 200 μg/mL. Regression equations were calculated using the equation *y = ax ± b,* where *y* and *x* are the peak areas and concentrations of the sample, respectively.

### 4.12. Preparation of the Main Components of ARAE

The main components of ARAE were identified using HPLC analysis, as described above. Each compound was dissolved in 100% DMSO. The stock concentration of the three compounds atractylenolide I, atractylenolide III, and atractylodin was 50 mM, and the intracellular concentrations applied were 1–100 μM (DMSO concentrations of 0.2% or less). The effects of these compounds on the viability of BV2 cells was measured, and their inhibitory effects on the secretion of NO and inflammatory cytokines induced by LPS was confirmed.

### 4.13. Statistical Analyses

All data are presented as means ± standard error of the mean (SEM) of three independent experiments unless stated otherwise. Statistical significance was determined using one-way analysis of variance followed by Dunnett’s test, after comparing each treated group and the LPS group. Each experiment was repeated three or more times, to yield comparable results. Values of * *p* < 0.05, ** *p* < 0.001, and *** *p* < 0.0001 were considered to indicate statistical significance.

### 4.14. Statistical Analysis Software

GraphPad Prism version 5.02 software (GraphPad Software, Inc., San Diego, CA, USA) was used for all the statistical analyses in this study.

## 5. Conclusions

Our results indicated that ARAE exerted anti-neuroinflammatory effects in vitro by inhibiting pro-inflammatory factors such as NO and inflammatory cytokines including TNF-α, IL-6, and IL-1β from LPS-stimulated BV2 microglia cells. In addition, ARAE effectively inhibited the synthetic enzyme iNOS and COX-2 expression of inflammatory factors in both protein and mRNA levels. These beneficial activities are associated with blockades of NF-κB and MAPK including ERK, p38, and JNK signaling pathways and enhanced HO-1 activation. Furthermore, given the inhibitory activities of the three constituents, the anti-neuroinflammatory effect of ARAE appears to be closely related to the presence of atractylenolide I, atractylenolide III, and atractylodin. Based on the above results, ARAE appears to have potential value as a candidate for the treatment of inflammation-associated neurodegenerative diseases.

## Figures and Tables

**Table 1:** Regression data and contents of three compounds in Atractylodis Rhizoma Alba ethanolic extract (ARAE).

Analytes	Regression Equation	*R* ^2^	Content (mg/g)
Atractylenolide I (**1**)	*y* = 0.0572*x* − 0.122	0.9998	16.04287 ± 0.0175
Atractylenolide III (**2**)	*y* = 0.7879*x* − 1.5886	0.9998	10.39714 ± 0.0712
Atractylodin (**3**)	*y* = 0.4807*x* − 1.1052	0.9998	17.06388 ± 0.0702

**Table 2:** Primers used for real-time reverse transcription-polymerase chain reaction (real-time RT-PCR).

Target Gene	Primer Sequence
*TNF-α*	F: 5′-TTCTGTCTACTGAACTTCGGGGTGATCGGTCC-3′
	R: 5′-GTATGAGATAGCAAATCGGCTGACGGTGTGGG-3′
*IL-6*	F: 5′-TCCAGTTGCCTTCTTGGGAC-3′
	R: 5′-GTGTAATTAAGCCTCCGACTTG-3′
*IL-1β*	F: 5′-ATGGCAACTGTTCCTGAACTCAACT-3′
	R: 5′-CAGGACAGGTATAGATTCTTTCCTTT-3′
*iNOS*	F: 5′-GGCAGCCTGTGAGACCTTTG-3′
	R: 5′-GCATTGGAAGTGAAGCGTTTC-3′
*COX-2*	F: 5′-TGAGTACCGCAAACGCTTCTC-3′
	R: 5′-TGGACGAGGTTTTTCCACCAG-3′
*HO-1*	F: 5′-TGAAGGAGGCCACCAAGGAGG-3′
	R: 5′-AGAGGTCACCCAGGTAGCGGG-3′
*β-actin*	F: 5′-AGAGGGAAATCGTGCGTGAC-3′
	R: 5′-CAATAGTGATGACCTGGCCGT-3′

F, forward; R, reverse.
